# Supplementary material for: Inhibition of Liver Metastasis in Colorectal Cancer by Targeting IL-13/IL13Rα2 Binding Site with Specific Monoclonal Antibodies
Source: Cancers (Basel). 2021 Apr 6;13(7):1731. doi: 10.3390/cancers13071731 (PMC8038733; doi:10.3390/cancers13071731)
Supplement: Supplementary file 1 [file cancers-13-01731-s001.zip › cancers-1159256-supplement.pdf]

cancers

MDPI

Supplementary Materials

Inhibition of Liver Metastasis in Colorectal Cancer by Targeting IL-13/IL13Rα2 Binding Site with Specific Monoclonal Antibodies

Marta Jaén, Rubén A. Bartolomé, Carmen Aizpurua, Ángela Martin-Regalado, J. Ignacio Imbaud and J. Ignacio Casal

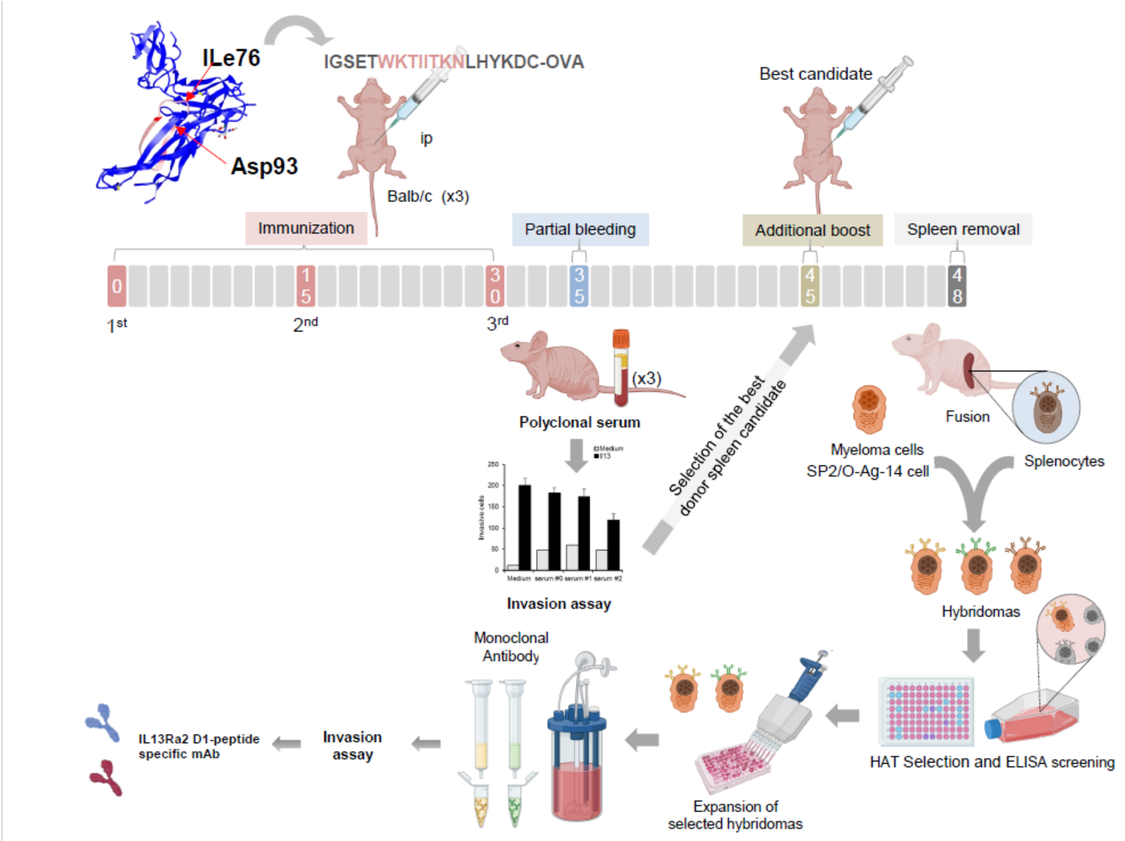

Figure S1: A representative scheme of the immunization and screening procedure for the generation of D1-specific monoclonal antibodies.

Table S1: List of antibodies used in the experiments.

**Antibody**	**Target**	**Application**	**Supplier**
ab55275	IL13Rα2	Flow cytometry	Abcam
PA5-46976	IL13Rα2	Flow cytometry	Thermo Fisher
clone 47	IL13Rα2	Blocking	Descibed in Balyasnikova et al. 2012 J. Biol. Chem. 287:30215-30227.
2K8	IL13Rα2	Immunoprecipitation, western blotting	Santa Cruz Biotechnologies
A-20	RhoGDIα	Western blotting	Santa Cruz Biotechnologies
D7A5	Phospho-Tyr1068 EGFR	Western blotting	Cell Signaling Technology
#4695	p44/42 MAP Kinase	Western blotting	Cell Signaling Technology
#9106	pThr202-p44/ pTyr204-p42 MAPK	Western blotting	Cell Signaling Technology
A-17	FAK	Western blotting	Santa Cruz Biotechnologies
FAK (pY397) 14	pTyr397-FAK	Western blotting	BD Transduction Laboratories
AF3389	SRC	Western blotting	R&D Systems
#6943	pTyr416 Src family	Western blotting	Cell Signaling Technology
#2105	pTyr530 Src	Western blotting	Cell Signaling Technology
#2920	AKT	Western blotting	Cell Signaling Technology
#3787	pSer473-AKT	Western blotting	Cell Signaling Technology
#9364	pSTAT6	Western blotting	Cell Signaling Technology
#9362	STAT6	Western blotting	Cell Signaling Technology
