# Supplementary figures and images for: Inhibition of Liver Metastasis in Colorectal Cancer by Targeting IL-13/IL13Rα2 Binding Site with Specific Monoclonal Antibodies
Source: Cancers (Basel). 2021 Apr 6;13(7):1731. doi: 10.3390/cancers13071731 (PMC8038733; doi:10.3390/cancers13071731)

Fig. 2 C

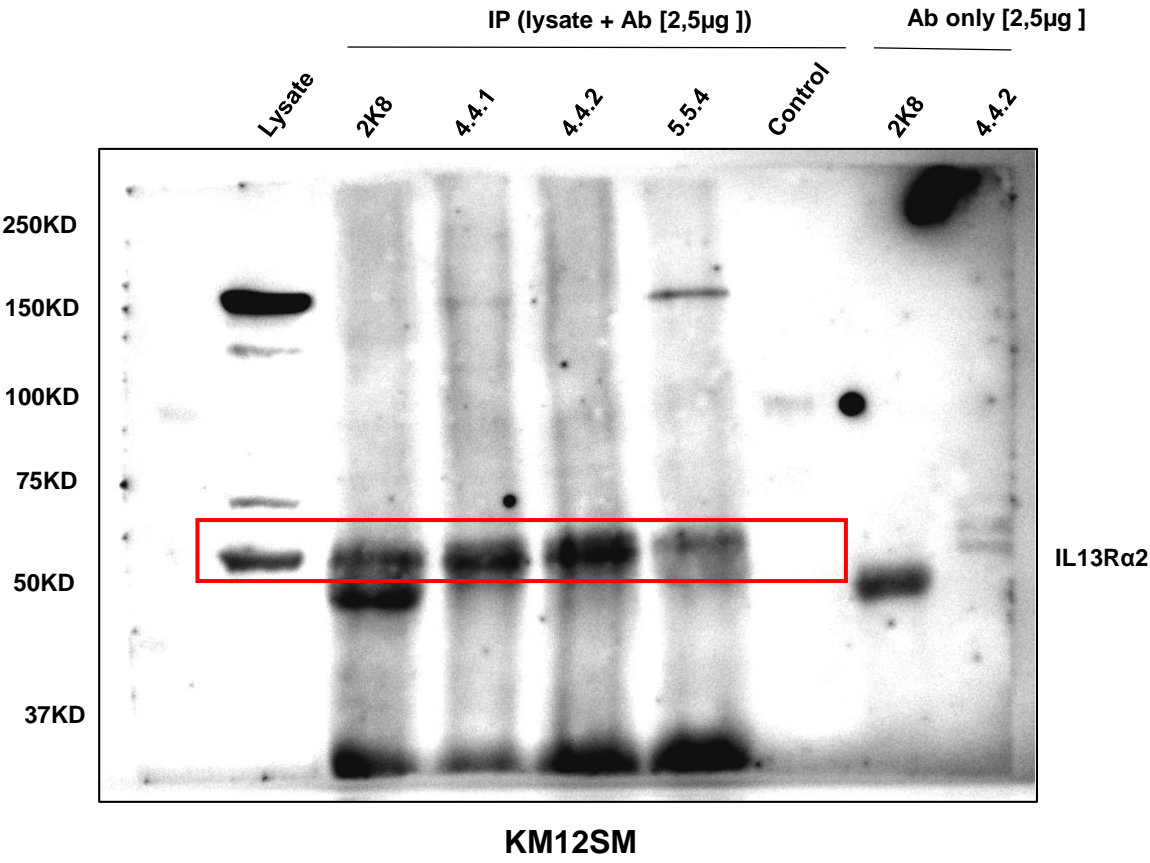

Fig. 4 KM12SM

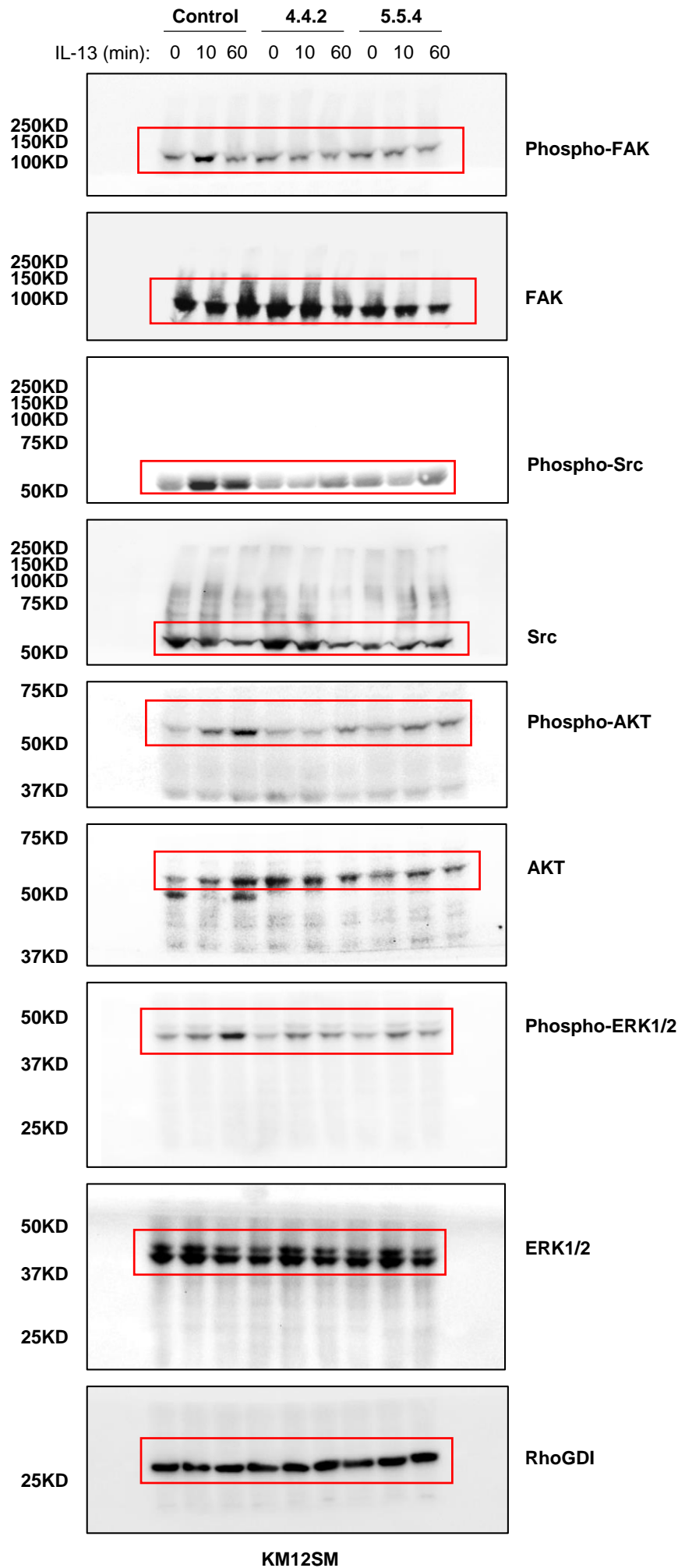

**Fig. 4 SW620**

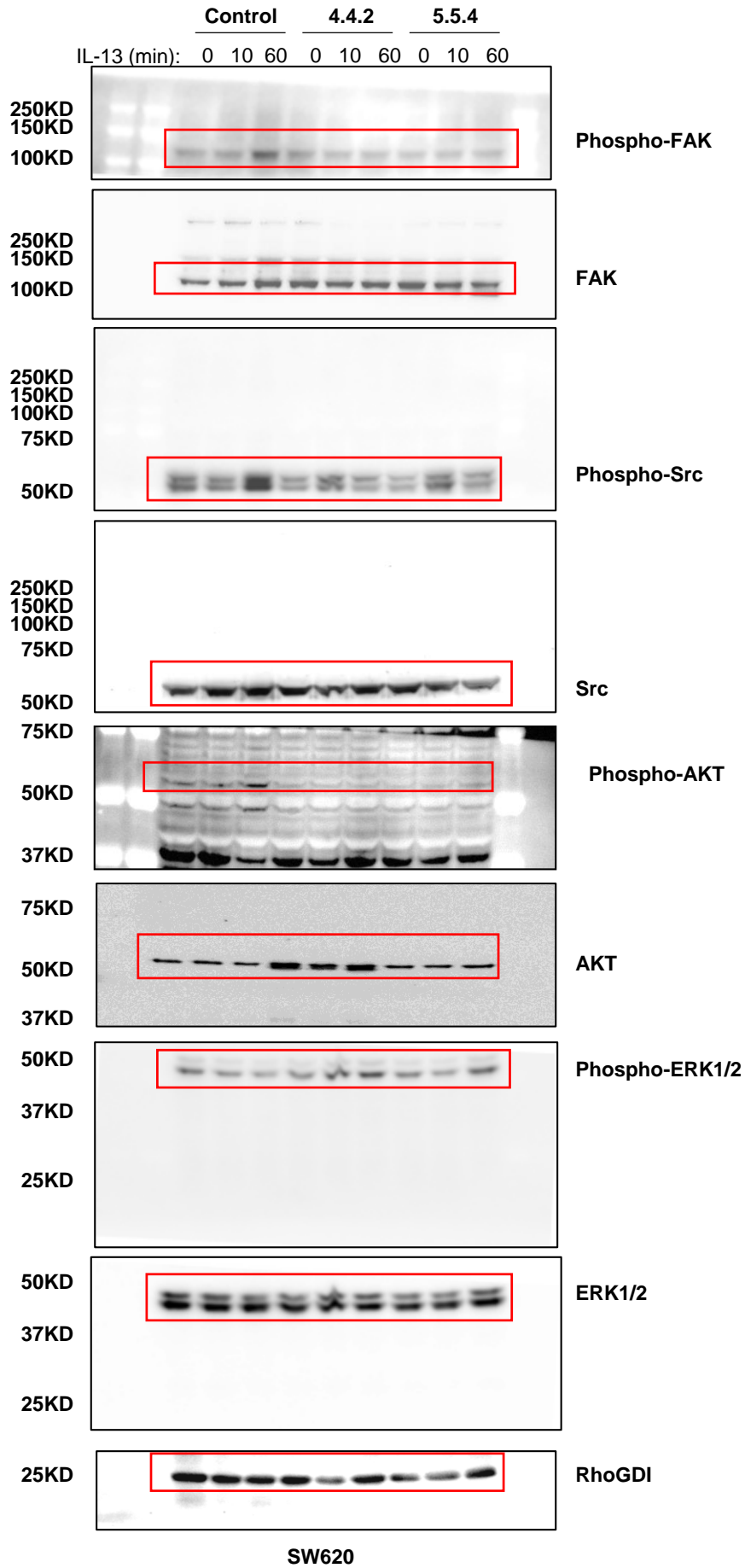

Fig. 4 RKO

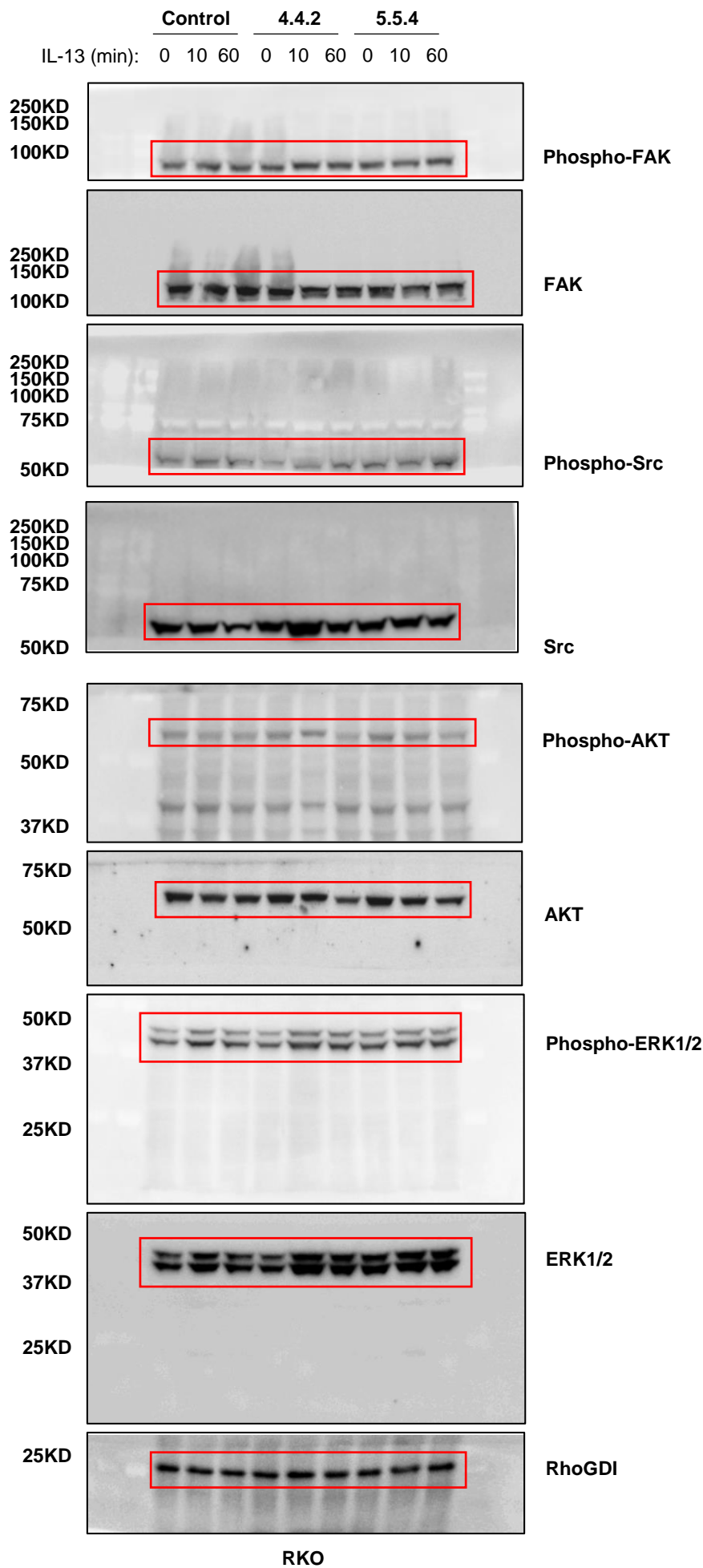

RKO

Fig. 4 CT26

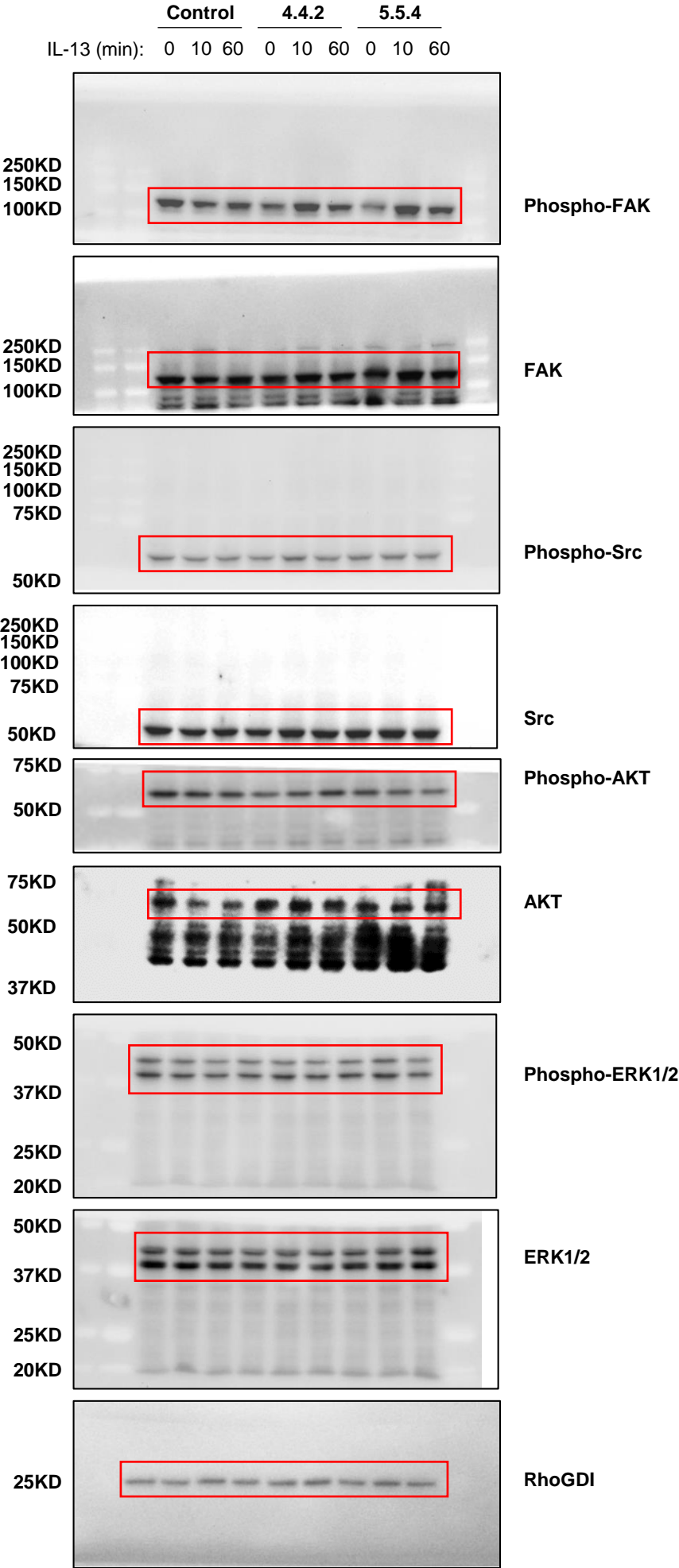

**Fig. 5 A**

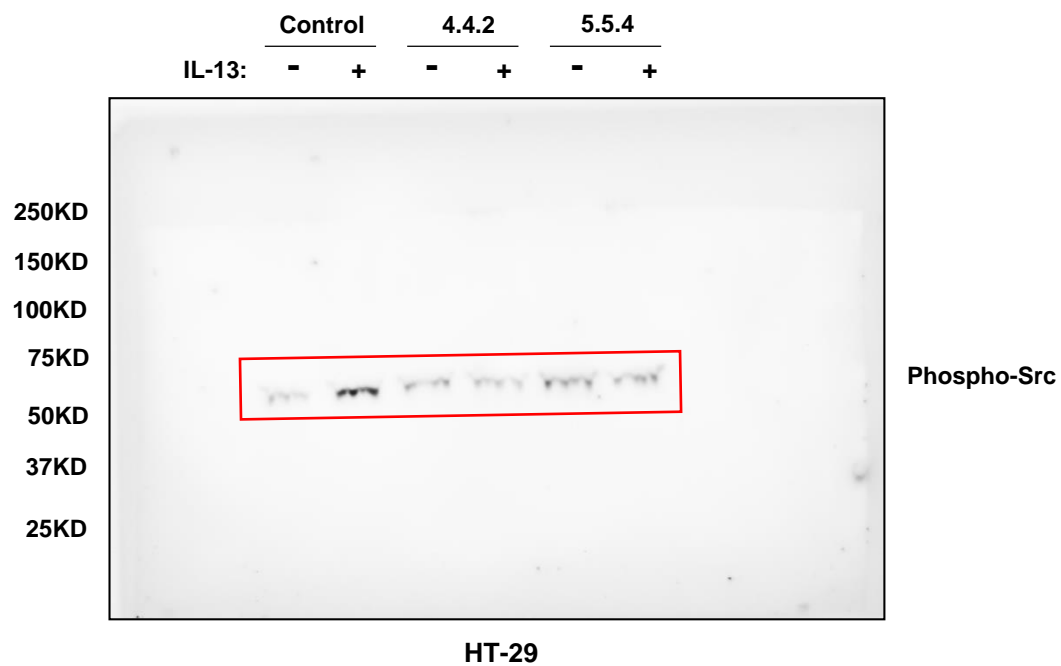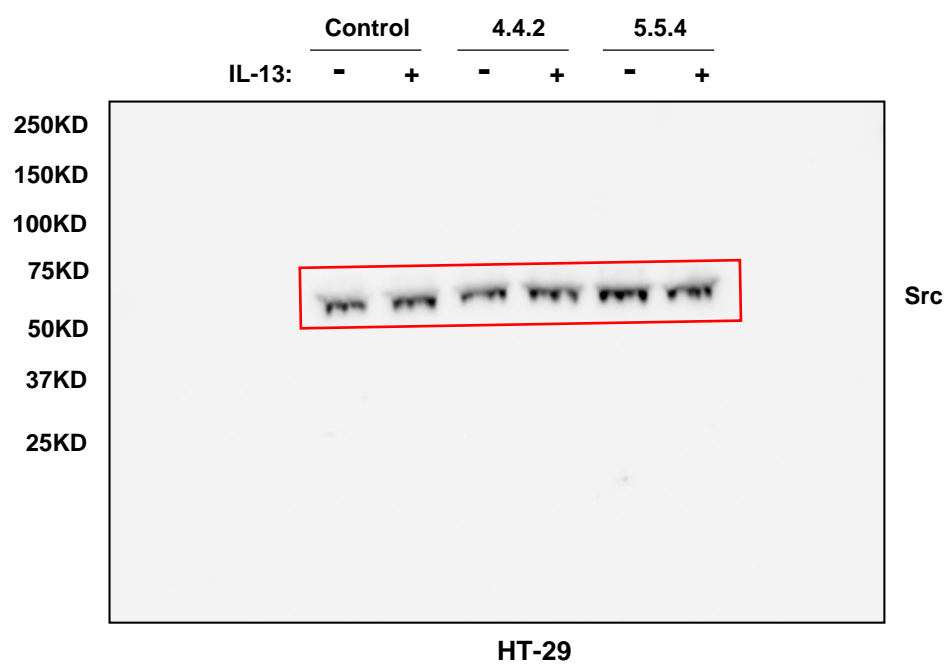

Fig. 5 A

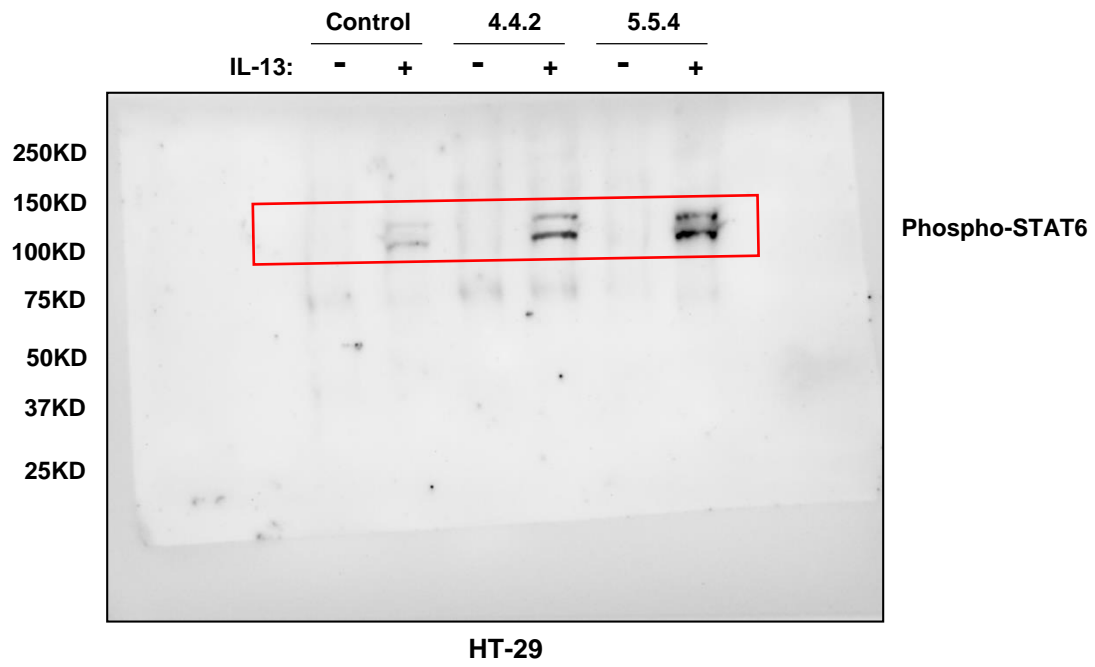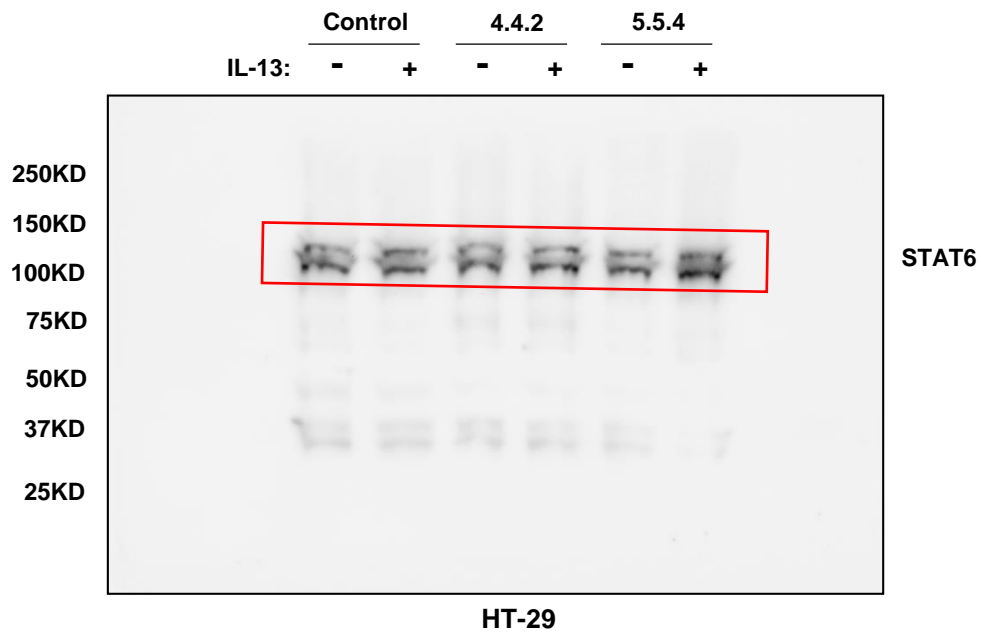

Fig. 5 C

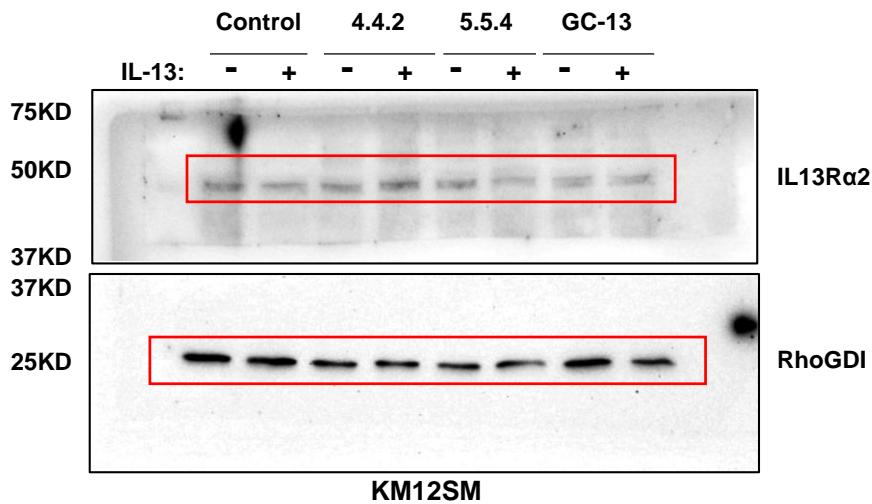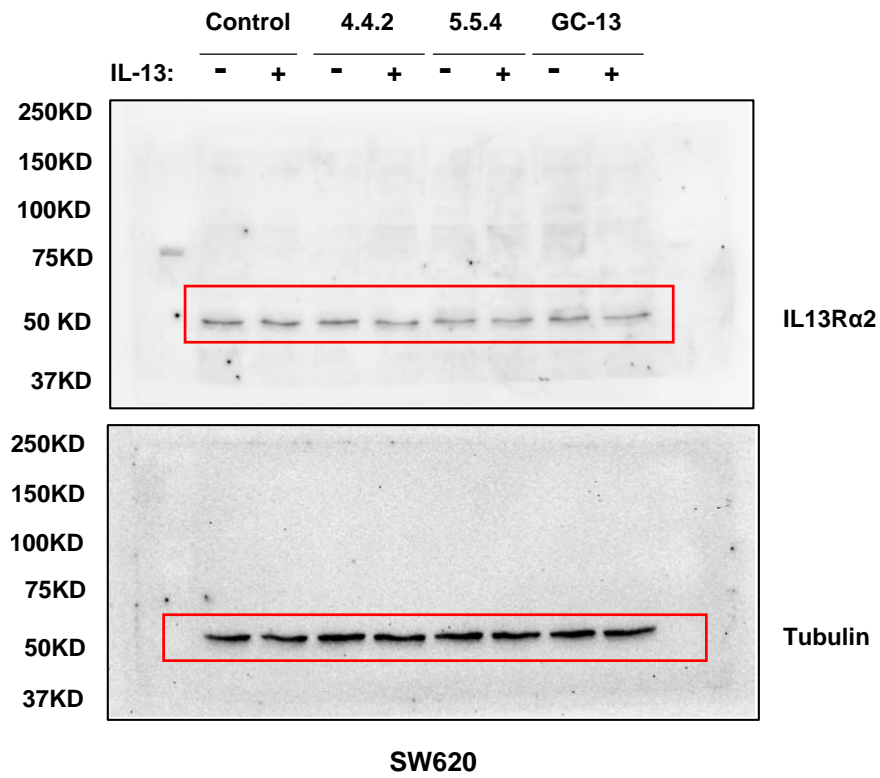

Supplement: Supplementary file 1 [file cancers-13-01731-s001.zip › cancers-1159256-Figure S2.pdf]
